# Interventions for the prevention of pain associated with the placement of intrauterine contraceptives: An updated review

**DOI:** 10.1111/aogs.13662

**Published:** 2019-06-27

**Authors:** Kristina Gemzell‐Danielsson, Jeffrey T. Jensen, Ilza Monteiro, Tina Peers, Maria Rodriguez, Attilio Di Spiezio Sardo, Luis Bahamondes

**Affiliations:** ^1^ Department of Women's and Children's Health Karolinska Institutet WHO‐Center Karolinska University Hospital Stockholm Sweden; ^2^ Department of Obstetrics and Gynecology Oregon Health & Science University Portland OR USA; ^3^ Department of Obstetrics and Gynecology Faculty of Medical Sciences University of Campinas Campinas Brazil; ^4^ Clare Park Hospital Farnham UK; ^5^ Department of Public Health School of Medicine University of Naples Federico II Naples Italy

**Keywords:** contraception, intrauterine contraception, intrauterine device, nonsteroidal anti‐inflammatory drugs, pain, women

## Abstract

A 2013 review found no evidence to support the routine use of pain relief for intrauterine contraceptive (IUC) placement; however, fear of pain with placement continues to be a barrier to use for some women. This narrative review set out to identify (1) new evidence that may support routine use of pain management strategies for IUC placement; (2) procedure‐related approaches that may have a positive impact on the pain experience; and (3) factors that may help healthcare professionals identify women at increased risk of pain with IUC placement. A literature search of the PubMed and Cochrane library databases revealed 550 citations, from which we identified 43 new and pertinent studies for review. Thirteen randomized clinical trials, published since 2012, described reductions in placement‐related pain with administration of oral and local analgesia (oral ketorolac, local analgesia with different lidocaine formulations) and cervical priming when compared with placebo or controls. Four studies suggested that ultrasound guidance, balloon dilation, and a modified placement device may help to minimize the pain experienced with IUC placement. Eight publications suggested that previous cesarean delivery, timing of insertion relative to menstruation, dysmenorrhea, expected pain, baseline anxiety, and size of insertion tube may affect the pain experienced with IUC placement. Oral and local analgesia and cervical priming can be effective in minimizing IUC placement‐related pain when compared with placebo, but routine use remains subject for debate. Predictive factors may help healthcare professionals to identify women at risk of experiencing pain. Targeted use of effective strategies in these women may be a useful approach while research continues in this area.

**Abbreviations**
IUC: intrauterine contraception
LNG‐IUS: levonorgestrel‐releasing intrauterine system
LP: lidocaine‐prilocaine
NSAID: nonsteroidal anti‐inflammatory drug
RCT: randomized clinical trial
VAS: visual analogue scale

**Key message**

Analgesia and cervical priming can be effective in reducing intrauterine contraception placement‐related pain when compared with placebo, but routine use remains a subject for debate. Targeted use of effective strategies in women identified as being at greater risk of experiencing pain may be a useful approach.

## 1 INTRODUCTION

Intrauterine contraceptives (IUC) are highly effective and safe methods of pregnancy prevention.^1^ Greater awareness and use could help to reduce the incidence of unplanned or mistimed pregnancy.^2^, ^3^ The US‐based Contraceptive CHOICE Project reported that provision of accurate, unbiased counseling on long‐acting reversible contraceptive methods, and removal of cost barriers, led to the majority of the women choosing IUC.^2^ A subsequent review of the impact of the CHOICE project showed that use of long‐acting reversible contraceptive methods led to higher rates of continued contraceptive use and a reduction in the average annual rates of pregnancy, birth and abortion among teenage participants.^3^

For most women, pain experienced with IUC placement is mild to moderate and less than anticipated.^4^, ^5^ However, some women remain anxious about the possibility of pain or are more likely to be affected by factors such as nulliparity, or a long time period since delivery. In addition, anatomical, cultural or psychological elements can contribute to a more painful experience.^6^, ^7^

Fear of pain at the time of placement can therefore be a barrier to choosing IUC.^6^, ^8^, ^9^ In a survey of pain and discomfort, both at the time of IUC placement and as a recollection, experienced by parous and nulliparous women participating in a UK‐based contraceptive service, Murty^9^ found that women anticipating pain were more anxious and more likely to take analgesia before placement. Although their pain scores during the procedure were similar to those of women who had not taken analgesia, their recollection of the pain experienced when asked 6 months later was greater than they reported immediately post‐placement. Healthcare professional concerns about difficult and/or painful placement may also discourage discussion of IUC as a contraceptive option and lead to the counseling of women on other, less effective methods.^10^

A literature review to evaluate the evidence for strategies to minimize pain experienced during IUC placement, carried out in 2012, led to a consensus that no prophylactic pharmacological intervention had been adequately studied to support its routine use.^11^ Furthermore, in a Cochrane Review of interventions to minimize pain associated with IUC placement, the authors concluded that some oral analgesics and lidocaine formulations are effective in reducing placement‐related pain in specific groups but that most of the evidence came from single trials and were of moderate quality.^8^ We undertook an updated review to determine whether there was any new evidence for pharmacological interventions to minimize pain associated with IUC placement, to identify whether nonpharmacological or procedure‐related interventions could prove helpful. We also set out to identify any factors that may assist in predicting the women most likely to experience pain.

## 2 MATERIAL AND METHODS

The broader objectives of this review, together with the diversity of populations and interventions and lack of consistent and validated assessment of pain experience, limits the possibility of a systematic review or meta‐analysis.^12^ Consequently, we opted to undertake a narrative review, an approach that is based on systematic methodologies, employs a bibliographic research strategy,^13^, ^14^ and looks at the evidence contributing to the clinical concept of pain relief during IUC placement.

We searched the PubMed and Cochrane databases to update and extend the findings of our original review.^11^ We conducted a search for publications in any language that reported pharmacological interventions to reduce pain with IUC placement using the terms “intrauterine contraception” AND “insertion” AND “pain” published from December 2012 to September 2018. We then conducted a search of publications from January 1980 to September 2018, the timeframe of the original and our updated review combined, to identify other approaches that may influence pain experience or predict the experience of pain. We used a combination of text and MeSH terms: “intrauterine contraception” OR “levonorgestrel‐releasing intrauterine system” OR “LNG‐IUS” OR “IUD” AND “pain” OR “anxiety” OR “fear” OR “counseling” OR “insertion” OR “placement” OR “initiation” OR “cervical priming” OR “cervical ripening.” The search was not limited to randomized controlled trials and results were cross‐referenced and duplicate publications were removed.

**Figure 1:**
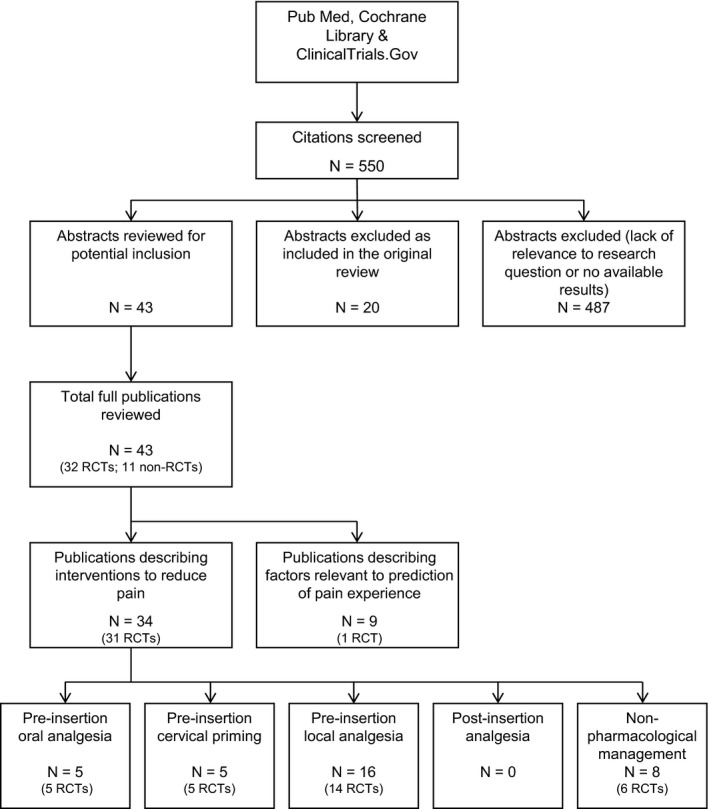
Literature selection process for review

The search identified 550 publications (Figure 1). Those not relevant to pain management either before or after IUC placement; those included in the previously published review^11^; and those reporting the findings of reviews were excluded. The final number of publications included in our review was 43. We assessed these publications for information relating to the effect of pharmacological interventions (pre‐insertion oral or local analgesia, cervical priming, post‐insertion analgesia), nonpharmacological strategies or procedure‐related factors on the pain experienced with IUC placement. We also assessed publications for factors that may help to predict the likelihood of experiencing pain with placement. Assessment of risk of bias of R randomized clinical trials (RCTs) included in the review was carried out according to the *Cochrane Collaboration Handbook for Systematic Reviews of Interventions*.^15^ As the intention was to conduct a narrative review rather than a systematic review the assessment of bias was limited to RCTs. No non‐English publications were identified for inclusion.

## 3 RESULTS

Information regarding study participants, intervention, method of pain evaluation, comparison of pain scores during IUC placement between the intervention and placebo/control groups, and level of evidence from each study reporting pain‐relieving interventions is summarized in Table 1. The clinical relevance and statistical significance of differences in pain scores between the intervention and placebo/control groups are also noted. There is a degree of overlap across the study descriptions as some studies described multiple interventions relevant to more than one category.

**Table 1:** Studies of pre‐placement pharmacological interventions for reduction of pain associated with IUC placement

Author(s)	N	Population	Type of study	Interventions	Method of evaluation of pain^a^	Effect of intervention vs placebo/control on pain score during IUC placement procedure	Level of evidence^c^
Pre‐insertion oral analgesia							
Crawford et al^16^	72	Mainly parous	RCT	20 mg ketorolac vs placebo	Multiple time points; 0‐10 cm VAS	Reduction in mean pain scores vs placebo at IUC placement (4.2 vs 5.7 cm, *P* = 0.031). Reduction in overall pain (3.6 vs 4.9 cm, *P* = 0.047) and pain 10 min post‐procedure (1.1 vs 2.5, *P* = 0.007)	2
Ngo et al^17^	118	Mainly nulliparous	RCT	550 mg naproxen sodium vs placebo	Multiple time points; 0‐100 mm VAS	No difference in median pain score vs placebo at IUC placement (69 vs 66 mm, *P* = 0.89). Reduction in median pain scores at 5 min (17 vs 26.0 mm, *P* = 0.01) and at 15 min (13 vs 24.0 mm, *P* = 0.01) post‐placement.	2
Singh et al^18^	80	Nulliparous	RCT	N_2_O/O_2_ vs no pain relief	Measured at time of placement on 0‐100 mm VAS	No difference in mean pain score vs control at IUC placement (54.3 ± 24.8 mm vs 55.3 ± 20.9 mm, *P* = 0.86)	2
Bednarek et al^19^	202	Nulliparous and parous	RCT	Ibuprofen vs no pain relief	Multiple time points; 0‐100 mm VAS	No difference in median pain score vs placebo at IUC placement (38.0 mm vs 41.5 mm, *P* = 0.5)	2
Castro et al^20^	100	Only cesarean delivery and nulligravidas	RCT	NSAID (400 mg ibuprofen) vs 2% lidocaine intracervical injection	Multiple time points; 0‐100 mm VAS	No difference in mean pain score vs lidocaine injection at IUC placement (48.1 ± 27.5 mm vs 44 ± 24.5 mm, *P* = 0.4)	2
Pre‐insertion cervical priming							
Maged et al^21^	120	Cesarean delivery	RCT	600 μg misoprostol vs placebo	Measured at time of placement on 0‐10 cm VAS	Reduction in mean pain score vs placebo at IUC placement (5.7 ± 1.4 cm vs 6.5 ± 0.9 cm)	2
Espey et al^23^	82	Nulliparous	RCT	400 μg misoprostol vs placebo	Measured at time of placement on 0‐10 cm VAS	No difference in mean pain score vs placebo at IUC placement (5.8 ± 2.0 cm vs 5.9 ± 2.0 cm, *P* = 0.94)	2
Abdellah et al^24^	140	Cesarean delivery	RCT	800 μg misoprostol vs placebo	Measured at time of placement on 0‐10 cm VAS	Reduction in mean pain score vs placebo at IUC placement (2.7 ± 0.6 cm vs 4.3 ± 0.8 cm, *P* = 0.001)	2
Scavuzzi et al^25^	179	Nulliparous	RCT	400 μg misoprostol vs placebo	Multiple time‐points; VAS 0‐10 cm (analyzed as absent or mild [0‐5] vs moderate or severe [6‐10]	Reduction in moderate or severe pain score vs placebo at IUC placement (OR 0.30, 95% CI 0.16‐0.55)	2
Lathrop et al^26^	73	Nulliparous	RCT	400 μg misoprostol vs placebo	Multiple time points; 0‐100 mm VAS	Increase in mean pain score vs placebo immediately prior (10.84 vs 2.11 mm; *P* = 0.005) and post‐placement (46.5 vs 35.4 mm, *P* = 0.04)	2
Pre‐insertion local anesthesia							
Mody et al^27^	64	Nulliparous	RCT	20‐mL 1% buffered lidocaine paracervical block vs placebo	Multiple time‐points; 0‐100 mm VAS	Reduction in median pain score vs placebo at IUC placement (33 vs 54 mm, *P* = 0.002)^b^. Reduction in median pain scores at other time‐points: uterine sounding (30 vs 47 mm, *P* = 0.005); 5 min after placement (12 vs 27 mm, *P* = 0.005); overall pain perception (30 vs 51 mm, *P* = 0.015). Increased pain score with block administration vs placebo (30 vs 8 mm, *P* = 0.003)	
Conti et al^28^	220	Nulliparous and parous	RCT	20 mL of 2% lidocaine gel self‐administered ≤ 15 min prior to IUC placement vs placebo gel	Multiple time‐points; 0‐100 mm VAS	No difference in median pain score vs placebo at IUC placement (65 [1‐99] mm vs 59 [5‐100] mm, *P* = 0.09). Reduction in median pain scores at speculum placement vs placebo (7 (0‐81) mm vs 11 (0‐80) mm, *P* = 0.046)	
Abd Ellah et al^29^	48	Nulliparous and parous	RCT	Self‐inserted novel lidocaine dual‐response in situ gel vs placebo	Multiple time‐points; 0‐10 cm VAS	Reduction in mean pain scores vs placebo at IUC placement (median [IQR]:3[2‐3.75] cm vs 6[5.5‐7] cm, *P* = 0.0001)^b^. Reduction in median pain scores at other time‐points: tenaculum placement (median [IQR]: 2[1‐2] cm vs 4[3‐4] cm, *P* = 0.0001); uterine sound insertion (median[IQR]: 3[2‐3] cm vs 5[4‐6] cm, *P* = 0.0001)	2
Akers et al^30^	95	Nulliparous	RCT	1% lidocaine paracervical block vs a sham control (1 cm depression of the vaginal epithelium at paracervical block sites with a wooden cotton‐tipped applicator)	Multiple time‐points; 0‐100 mm VAS	Reduction in mean pain score vs sham block at IUC placement (30.0 [95% CI 20‐58] mm vs 71.5 [95% CI 66.0‐82.0] mm, *P* = 0.006)^b^. Analysis of the secondary outcomes found that the scores across all six VAS assessments (baseline, speculum, tenaculum, block, uterine sounding, IUC placement, and speculum removal were lower in the lidocaine block group compared with the sham block group (27.7 [95% CI 16.0‐40.2] vs 53.9 [95% CI 44.0‐57.8], *P* < 0.001)	2
Karasu et al^31^	151	Parous	RCT	Lidocaine spray (10%, net 40 mg) vs 2 g lidocaine cream vs 10 mL lidocaine injection (20 mg/mL)	Tenaculum and IUC placement; 0‐10 cm VAS (categorized as none [0], mild [1‐3], moderate [4‐6], severe [7‐10])	Reduction in mean pain score vs LP cream and control (no anesthesia) at IUC placement (2.85 ± 2.5 cm vs 4.0 ± 1.7 cm vs 4.25 ± 1.9, respectively). Greater proportion of women experiencing no pain at IUC placement with spray vs cream, injection and control groups (25.5% vs 1.9% vs 2.1% vs 2.0%, respectively, *P* = 0.01) and at tenaculum placement (39.2% vs 7.5% vs 6.4% vs 4.1%, respectively, *P* = 0.01)	2
Abbas et al^32^	120	Parous	RCT	Lidocaine‐prilocaine cream (LP) vs placebo	Multiple time‐points; 0‐10 cm VAS	Reduction in median pain score vs placebo at IUC placement (3.0 vs 6.5 cm, *P* = 0.0001)^b^	2
Torky et al^41^	420	Parous	Non‐randomized comparator study	Lidocaine gel or lidocaine spray vs no anesthesia	Multiple time‐points; 0‐10 cm VAS	No difference in mean pain score at IUC placement: lidocaine gel vs lidocaine spray vs no anesthesia (4.9 ± 1.9 cm vs 4.6 ± 1.9 cm vs 5.9 ± 1.5 cm respectively, *P* = 0.059). Reduction in mean pain score during cervical traction with use of local anesthesia (4.5 ± 2.1 cm for lidocaine gel; 4.0 ± 2.2 cm for lidocaine spray vs 6.0 ± 1.5 cm for no anesthetic, *P* = 0.003)	2
Elkhouly and Maherl^21^	200	Mostly nulliparous	RCT	1% lidocaine intracervical block, misoprostol (400 μg), oral naproxen (500 mg) vs placebo	Multiple time‐points; 0‐10 cm VAS	No difference in mean pain score vs placebo at IUC placement with lidocaine block vs misoprostol, oral naproxen and placebo (5.3 ± 1.61 cm, 5.02 ± 1.12 cm, 4.9 ± 1.24 vs 5.16 ± 1.21 cm, respectively, *P* = 0.460)	2
Rapkin et al^33^	59	Nulliparous	RCT	Self‐administered 2% lidocaine gel vs placebo	Multiple time‐points; 0‐100 mm VAS	No difference in median pain score vs placebo at IUC placement (61 mm [IQR 53‐71]) vs (69 mm [IQR 63‐80], *P* = 0.06). Reduction vs placebo at tenaculum placement (32 [IQR 18‐54] mm vs 56 [IQR 26‐75] mm, *P* = 0.02)	2
Fouda et al^34^	90	Parous	RCT	Diclofenac potassium (2 × 50 mg 1 h prior) plus application of 3 mL 2% lidocaine gel on the cervical lip and cotton swab soaked in 2% lidocaine gel in the cervical canal (3 min prior) vs placebo	Multiple time‐points; 0‐10 cm VAS	Slight reduction in mean pain score vs placebo (3.14 ± 0.92 cm vs 3.94 ± 1.3 cm, *P* = 0.001)	2
Aksoy et al^35^	200	Parous	RCT	Lidocaine spray (10%, net 40 mg) vs placebo	Measured at time of placement on 0‐10 cm VAS	Reduction in mean pain score vs placebo at IUC placement (1.01 ± 1.20 cm vs 3.23 ± 1.60 cm, *P* < 0.001)^b^. Decrease in the proportion of women scoring ≥ 4 on the pain‐VAS vs placebo (6% vs 41%, *P* < 0.001)	2
Tavakolian et al^36^	92	Parous	RCT	Lidocaine‐prilocaine cream (LP) vs placebo	Multiple time‐points; 0‐10 cm VAS (categorized as none [0 points], mild [1‐3], average [4‐6] and severe [9], extremely severe [10]	Reduction in mean pain score vs placebo at IUC placement (2.65 ± 2.53 cm vs 4.61 ± 2.55 cm, *P* < 0.001)^b^	2
Tornblom‐Paulander et al^37^	218	Nulliparous	RCT	Lidocaine (8.5 ml) of a novel formulation (SHACT) vs placebo	Multiple time‐points; 0‐100 mm VAS (starting at 10 min post‐placement)	Reduction in maximum pain score at IUC placement vs placebo at 10 min (28.3 ± 24.6 mm vs 44.2 ± 26.0 mm, *P* < 0.0001)	2
Micks et al^38^	24	Nulliparous	RCT	0.5 mg nitroglycerin gel (1 mL) vs placebo gel	Multiple time‐points; 0‐100 mm VAS	No difference in mean pain scores vs placebo (55.0 ± 29.7 mm vs 57.4 ± 22.1 mm, *P* = 0.82)	2
Allen et al^39^	145	Mostly parous	RCT	2% lidocaine gel vs placebo	Measured at time of placement on 0‐10 cm VAS	No difference in mean pain scores vs placebo (35.2 ± 27.7 mm vs 36.7 ± 30.0 mm, *P* = 0.8)	2
Nelson, Fong^40^	40	Nulliparous and parous	RCT	1.2% lidocaine infusion (via endometrial aspirator) vs saline	Multiple time‐points; 0‐9 scale (where 0 = no pain and 9 = worst pain imaginable)	No difference in mean pain scores vs placebo (2.95 vs 3.75, *P* = 0.37)	2

VAS, visual analogue scale; CI, confidence interval; OR, odds ratio.

a For studies in which pain was evaluated at different time‐points, the time‐points included one or more of the following: at speculum insertion, at tenaculum placement, during device placement, shortly after device placement (10 min) and longer intervals after placement (1 h, 2 h, 1 d, 2 d, and 3 d).

b Difference in pain scores between intervention and control/placebo regarded as clinically relevant according to Olsen et al.^61^

c Oxford Center for Evidence‐Based Medicine^60^ levels of evidence: Level 1, systematic review of randomized trials; Level 2, randomized trial or observational study with dramatic effect; Level 3, non‐randomized controlled cohort/follow‐up study; Level 4, case series, case‐control studies or historically controlled studies; Level 5, mechanism‐based reasoning.

### 3.1 Characteristics and methods of included studies

Among the reviewed publications, 26 described pre‐placement pharmacological interventions to minimize pain associated with IUC placement.^16^, ^17^, ^18^, ^19^, ^20^, ^21^, ^22^, ^23^, ^24^, ^25^, ^26^, ^27^, ^28^, ^29^, ^30^, ^31^, ^32^, ^33^, ^34^, ^35^, ^36^, ^37^, ^38^, ^39^, ^40^, ^41^ Of these, 25 were RCTs^16^, ^17^, ^18^, ^19^, ^20^, ^21^, ^22^, ^23^, ^24^, ^25^, ^26^, ^27^, ^28^, ^29^, ^30^, ^31^, ^32^, ^33^, ^34^, ^35^, ^36^, ^37^, ^38^, ^39^, ^40^ and one was a nonrandomized comparator study.^41^ Eight publications—of which six described RCTs,^42^, ^43^, ^44^, ^46^, ^47^, ^48^ one was a pilot feasibility study^45^ and one was a pooled analysis^49^described nonpharmacological interventions. A further nine publications described factors related to the experience of pain with IUC placement.^50^, ^51^, ^52^, ^53^, ^54^, ^55^, ^56^, ^57^, ^58^ Of these, one was an RCT,^50^ three were non‐RCTs,^51^, ^52^, ^53^ three were prospective cohort studies,^54^, ^55^, ^56^ one was a case‐control study,^57^ and one was a secondary analysis of the US‐based Contraceptive CHOICE Project.^58^

All studies evaluating pharmacological interventions except for Nelson and Fong^40^ reported the use of a 10‐cm or 100‐mm visual analogue scale (VAS), where 0 is equivalent to “no pain” and 10 is equivalent to “worst pain ever”, by study participants to indicate the severity of pain experienced. Nevertheless, there were wide variations in the assessment of the experience of pain in terms of timing (IUC placement only); IUC placement plus other time‐points (speculum insertion, tenaculum placement, uterine sounding); post‐placement assessment (multiple time intervals); overall perception of pain.

### 3.2 Pre‐insertion pharmacological therapy: Oral analgesia

Five RCTs evaluated the effectiveness of oral analgesia on the experience of pain with IUC placement^16^, ^17^, ^18^, ^19^, ^20^ and one RCT compared multiple analgesic agents.^21^ One RCT, reported a 15‐mm reduction in mean pain score with oral ketorolac (20 mg) given 40‐60 minutes before IUC placement when compared with placebo.^16^ Although statistically significant, the authors suggested that the time required for the maximum analgesic effect of ketorolac (1‐2 hours after administration) may have affected the outcome. The effect of the intervention may have been more accurate, although less practical, if measured at a later time‐point.^16^ Four studies, evaluating 550 mg naproxen sodium,^17^ N_2_O/O_2_ 18 and ibuprofen compared with placebo,^19^, ^20^ found no difference in mean pain scores at the time of IUC placement between the treatment and control groups; however, a reduction in median pain score was observed at 5 minutes (9 mm) and 15 minutes (11.2 mm) after IUC placement in the naproxen sodium group when compared with placebo.^17^

### 3.3 Pre‐insertion pharmacological therapy: Cervical priming

Three RCTs found pain scores with IUC placement to be lower following vaginal administration of misoprostol when compared with placebo (Table 2).^22^, ^24^, ^25^ Using cut‐offs of “absent or mild (VAS score of 0‐5 cm)” and “moderate to severe (VAS score of 6‐10 cm)”, Scavuzzi et al^25^ reported a reduction in moderate‐to‐severe pain with 400 μg misoprostol administered 4 hours before IUC placement in nulliparous women (risk ratio 0.56 [32/86 vs 62/93]; 95% confidence interval (CI) 0.41‐0.75; *P* = 0.00008). Two further studies found misoprostol (600 μg 6 hours prior and 800 μg 3 hours prior, respectively) reduced mean pain scores at placement by 0.8‐1.6 cm compared with placebo in women with a history of only elective cesarean delivery.^22^, ^24^ Although the 1.6‐cm reduction in pain score with misoprostol compared with placebo was statistically significant, Abdellah et al^24^ highlighted multiple factors limiting the validity of their study: pain minimization by women after a successful procedure, time taken for misoprostol to take effect, the lack of availability of the 52 mg levonorgestrel‐releasing intrauterine system (LNG‐IUS), and a high insertion failure rate.^24^ Furthermore, the frequency of abdominal cramping was increased in the misoprostol group in all three studies.^22^, ^24^, ^25^ Espey et al^23^ evaluated 400 μg of misoprostol administered 2‐8 hours before IUC placement in nulliparous women and found there to be no difference in pain scores at placement between the treatment and placebo groups. Lathrop et al^26^ found there to be an 11‐mm increase in pain experienced with misoprostol compared with placebo.

**Table 2:** Predictive factors for increased potential for painful IUC placement^52^, ^53^, ^54^, ^56^, ^58^, ^62^

Physical factors detected during routine history or examination	Psychological and sociocultural factors
Low parity (1‐2 live births)	Number of years in education (≥7)
Longer interval between last birth and placement (>13 months)	Presence of mood disorders
Nonbreastfeeding at time of placement (irrespective of time since last birth)	History of sexual trauma
Presence of cervical resistance and pain	Previous negative reaction to vaginal examination
Uterine length	Previous placement reported as painful
Dysmenorrhea	Awareness of the potential for pain from a friend or family member
Multiple cesarean deliveries	Anticipation or expectation of pain
Menstruation (for nulligravidas)	Age (adolescence)
Difficulty or pain when using uterine sound	
Size of inserter	

### 3.4 Pre‐insertion local anesthesia

A total of 16 studies, of which 14 were RCTs, evaluated the effect of pre‐insertion local anesthesia on pain experienced with IUC placement.^27^, ^28^, ^29^, ^30^, ^31^, ^32^, ^33^, ^34^, ^35^, ^36^, ^37^, ^38^, ^39^, ^40^, ^41^ Interventions included lidocaine gel, lidocaine‐prilocaine (LP) cream, lidocaine intracervical block, lidocaine infusion, and nitroglycerin gel. Nine RCTs reported lower VAS pain scores in the treatment group compared with the control/placebo group.^27^, ^29^, ^30^, ^31^, ^32^, ^34^, ^35^, ^36^, ^37^, ^41^

#### 3.4.1 Lidocaine spray

Two RCTs reported lower placement‐related pain scores with lidocaine spray (net 40 mg) when compared with 2 g lidocaine cream or 10 mL lidocaine (20 mg/mL) injection^31^ and placebo.^35^ In the study by Karasu et al,^31^ a greater number of women in the lidocaine spray group reported no pain with placement compared with the injection and cream group (25.5% vs 1.9% vs 2.1%; *P* < 0.001). In the study by Aksoy et al,^35^ use of lidocaine spray reduced mean pain scores by 2.2 cm when compared with placebo (1.01 ± 1.20 vs 3.23 ± 1.60, *P* > 0.001). The authors also found a 35% reduction in the number of women scoring ≥ 4 on the 10‐cm VAS (6% vs 41%, *P* < 0.001).^35^ Neither study involved nulliparous women, nor did they assess pain at time‐points other than IUC placement, an observation noted by Aksoy et al^35^ as being useful in evaluating the delayed prostaglandin‐related cramping response that many women can experience. Karasu et al^31^ discussed the variability in dosing options for all three lidocaine preparations used in the study and the risks involved in increasing doses over and above those routinely used in gynecological practice. A nonrandomized study^41^ compared the effect of cervical lidocaine spray, cervical lidocaine gel and no topical anesthesia in 420 women: mean pain scores were 1‐1.5 cm lower with the use of lidocaine preparations compared with no anesthetic.^41^

#### 3.4.2 Lidocaine paracervical block

Three RCTs evaluated the effect of lidocaine paracervical block on pain experienced with IUC placement.^27^, ^30^, ^31^ Akers et al^30^ found there to be a 41.5 mm reduction in mean pain score when comparing injection of 1% lidocaine with a sham control in nulliparous women (*P* = 0.006). The authors highlighted limitations relating to the proportion of highly educated and insured women included in the study; the use of small IUC devices and the lack of blinding of study coordinators responsible for collecting participant data.^30^ Mody et al^27^ reported a 21 mm reduction in median pain score with 1% lidocaine block compared with placebo in nulliparous women (*P* = 0.002). However, there was an increase in pain associated with the lidocaine injection (30 vs 8 mm, *P* = 0.003).^27^ The study by Karasu et al^31^ described above found that 10 mL 2% lidocaine injection, a dose higher than that used in most clinics, led to a lower mean pain score at placement when compared with LP cream and control (no anesthesia) (2.9 ± 1.4 vs 4.0 ± 1.7 vs 4.25 ± 1.9) but no difference in the proportion of women experiencing no pain at placement (2.1% vs 1.9% vs 2.0%). Two RCTs comparing lidocaine paracervical block with nonsteroidal anti‐inflammatory drugs (NSAIDs) and placebo (400 mg ibuprofen and 500 mg oral naproxen/placebo, respectively) found no differences in mean pain scores during placement.^20^, ^21^

#### 3.4.3 Lidocaine‐prilocaine cream

Two studies^31^, ^32^ reported 1.9‐3.5 cm reductions on a 10‐cm VAS in mean pain scores at multiple time‐points during placement in parous women following application of LP cream when compared with the control group. In the study by Karasu et al,^31^ use of LP cream did not lead to lower pain scores at either tenaculum or IUC placement when compared with controls (no anesthesia).

#### 3.4.4 Lidocaine gel

Authors of two studies^29^, ^37^ reported lower placement‐related pain scores with novel formulations of lidocaine gel when compared with placebo. A single, 8.5‐mL dose of a formulation designed to minimize leakage and prolong presence in the target tissues (SHACT) reduced maximum pain by 16.1 mm when compared with placebo in >200 nulliparous women.^37^ Abd Ellah et al^29^ reported the findings of a small study (n = 48), involving self‐administration of a dual‐response, in situ lidocaine gel formulation in both parous and nulliparous women. Median pain scores were 2‐3 cm lower at all steps of IUC placement (including tenaculum placement and uterine sounding) with the lidocaine gel compared with placebo. The authors attributed the lower pain scores to the controlled release behavior of the gel demonstrated in rheological studies.^29^

Three RCTs^28^, ^33^, ^39^ and one non‐RCT^41^ reported no difference in median pain scores with IUC placement with 2% lidocaine gel compared with placebo.

#### 3.4.5 Other local anesthesia approaches

Two RCTs, one comparing the effect of infusion of 1.2 mL of 2% lidocaine solution, administered by endometrial aspirator with placebo^40^ and one comparing the effect of 0.5 mg nitroglycerin gel (1 mL) with placebo,^38^ found no differences in mean pain scores between the treatment and control groups.

### 3.5 Post‐insertion pharmacological therapy

No new or additional studies were found in the updated literature search.

#### 3.5.1 Non‐pharmacological pain management

The broader search of the literature yielded four studies evaluating nonpharmacological strategies that may indirectly help to reduce pain experienced with IUC placement.^42^, ^44^, ^45^, ^46^ A successful reduction in anxiety (but not pain) was reported with use of inhaled lavender compared with placebo.^42^ Also, Arsenijevic et al^44^ reported a reduction in uterine and cervical injury during priming, which may impact on pain experience, with the use of a continuous, controllable balloon dilator compared with the Hegar dilator in a three‐arm study involving 120 women. Authors of a pilot feasibility study of a novel suction cervical retractor reported a 15‐mm reduction in mean pain score compared with placebo at IUC placement.^45^ Use of ultrasound‐guided IUC placement led to a 2.6‐cm reduction in mean pain score compared with the traditional, non‐guided technique (2.4 vs 5.0, *P* < 0.001).^46^ However, the authors acknowledged that presence of a full bladder could add to the pain experienced with speculum insertion. It was also noted that the need for availability of equipment and sonographic knowledge when placing the probe may not be feasible in all clinics.

A pooled analysis from three phase II studies involving the use of a modified placement device (EvoInserter^®^, insertion tube diameter of 3.8 mm) for the low‐dose 13.5 mg LNG‐IUS suggested that the reduced diameter contributed to ease of placement and manageable pain.^49^ Additional post‐hoc analyses showed a significant association (*P *=* *0.0001) between the women's evaluation of pain on placement that was maintained following adjustment for age and parity. However, all data were retrospective and comparative data were not available across all three studies. As options for pain management and cervical dilation were left at the discretion of the investigators, conclusions regarding their impact on ease of placement and experience of pain are not possible.^49^ One of the studies that contributed to this pooled analysis reported a greater proportion of women experiencing “no pain” or “only mild pain” during placement in a randomized comparison of the EvoInserter^®^ with the standard 52 mg LNG‐IUS with a larger (4.75 mm) insertion tube (72.3% vs 57.9%,).^59^

Two further RCTs found no difference in mean pain scores between vulsellum and a single‐tooth tenaculum^43^ or between tenaculum and Littlewood forceps^47^ when stabilizing the cervix for IUC placement. A comparison of delayed vs immediate emptying of a pre‐filled bladder also failed to show any significant difference in mean pain scores.^48^

### 3.6 Predictors of the pain experience

Eight publications described factors that may help to predict women more likely to experience pain with IUC placement.^50^, ^51^, ^52^, ^53^, ^54^, ^55^, ^56^, ^57^, ^58^ The identified factors are summarized in Table 2. Chi et al^57^ suggested that higher education (>7 years), low parity (1‐2 live births), a longer interval between birth and placement (>13 months), and nonbreastfeeding at time of insertion were all associated with severe pain on IUC insertion. Goldstuck and Matthews^51^ reported an increase in pain experienced in women with greater cervical resistance when measured using a Salter Abbey electronic force gauge with a modified recording probe into which the IUC was inserted. The authors also reported that expected pain was higher than immediate pain experienced by study subjects (*P* = 0.001).

Newer studies identified several additional factors that may exert a role in the experience of pain: timing of insertion relative to menstruation, previous cesarean delivery, history of dysmenorrhea, expected pain, baseline anxiety, and size of insertion tube.^50^, ^52^, ^53^, ^54^ One RCT^50^ compared pain with IUC placement in both nulliparous and parous women within days 1‐7 of menstruation and at any day without vaginal blood loss and found no difference in pain experienced. A non‐randomized study investigating menstrual and gynecologic history as predictors of difficult or painful IUC placement reported severe dysmenorrhea as the only predictor of placement‐related pain.^52^ Santos et al^53^ compared pain experienced during IUC placement in three group of women; nulligravidas, parous with vaginal delivery and parous only with cesarean delivery. Women who had undergone cesarean delivery were at greater risk of pain (moderate to severe [VAS score 4‐10 cm] experienced by 88%) than women in the other two groups, potentially due to an anatomically distorted uterus caused by hypertrophic scarring.^53^

Additionally, a linear regression analysis of the placebo‐controlled study evaluating the effect of high‐dose lidocaine gel on pain with IUC placement found nulliparity, interval IUC placement and a history of dysmenorrhea to be predictive of pain.^39^ In a multivariable analysis of the results of a prospective cohort study comparing pain with IUC placement in women with and without the experience of vaginal delivery by the same authors,^54^ a history of vaginal delivery was associated with a 15.5‐point reduction in mean pain score when compared with no previous vaginal delivery (*P* = 0.009). Other predictors of pain were “expected pain” and “placement difficulty”.^54^

A prospective cohort study^56^ found that the mean post‐placement pain experienced by nulliparous adolescents was higher than that among parous adult women on each day of the 2‐week study (*P* < 0.05) and the greatest mean difference occurred in the first 4 days. The authors suggested that pain scores may have been affected by the detailed pre‐ and post‐placement counseling of adolescent women provided by a trained Pediatric and Adolescent Gynecologist, which is uncommon in many settings and, therefore, different when compared with routine practice.^56^ There was also a recommendation to use ibuprofen by one professional responsible for this arm of the study and eight adolescents had the placement procedure under sedation.^56^

A secondary analysis of 1149 participants in the US Contraceptive CHOICE Project looked at whether anticipated pain affected actual pain experienced during IUC placement.^58^ After controlling for parity, history of dysmenorrhea and type of IUC, higher anticipated pain was associated with an increase in experienced pain (adjusted risk ratio for 1 unit increase in anticipated pain, 1.19; 95% CI 1.14‐1.25).^58^ Nulliparity, history of dysmenorrhea, and placement of an LNG‐IUS (with a 4.8‐mm inserter) were all associated with an increase in mean pain score with IUC placement. As CHOICE was a prospective cohort study, real‐time collection of anticipated and actual pain data limits recall bias and strengthens the study.^58^ However, lack of information regarding the use of pain‐relieving interventions, other than the routine offering of premedication with NSAIDs, may be a limiting factor.

A prospective cohort study by Narayan et al^55^ also looked at anticipated and actual pain related to IUC compared with implant placement. Although women choosing IUC had been told to expect the procedure to be painful, anticipated pain with both methods was similar. When asked about actual pain experienced via the post‐visit survey, women choosing IUC (n = 50) reported greater levels of pain than expected (7.0 vs 6.0, *P* = 0.004); whereas women choosing an implant reported less actual pain (2.0 vs 5.0, *P* = 0.001).^55^

### 3.7 Assessment of risk of bias and quality of included studies

**Figure 2:**
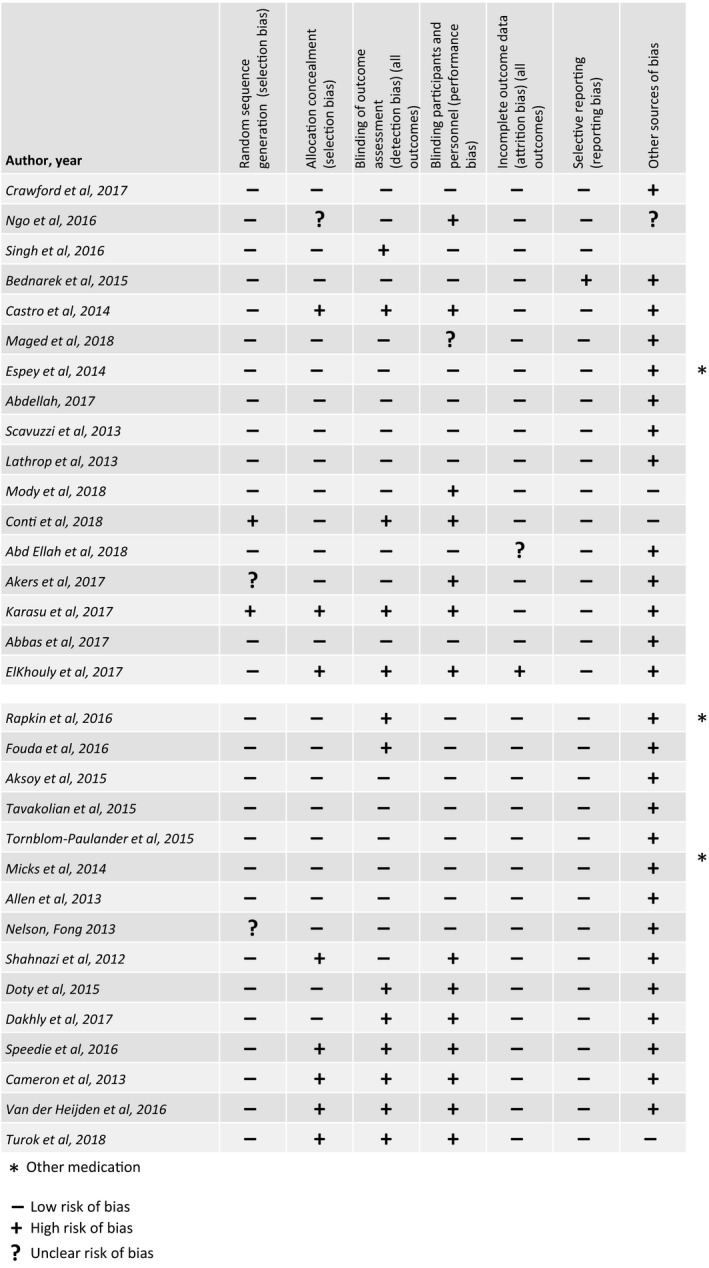
Assessment of risk of bias of RCTs [Color figure can be viewed at http://www.wileyonlinelibrary.com]

Each RCT included in the review was assessed for selection, performance, detection, attrition, and reporting bias by three authors (two assessors and one moderator) and the results are shown in Figure 2. The majority of the remaining non‐RCTs identified during our review described factors that may help identify those women at increased risk of pain with IUC placement. We did not assess these publications for risk of bias as their purpose was to provide insights into the potential for greater experience of pain than healthcare professionals may consider when counseling women regarding IUC. Given the nature of the review, ie narrative rather than systematic, assessment of quality of the RCTs did not extend beyond categorization according to the guidance of the Oxford Center for Evidence‐Based Medicine.^60^

Although 12 RCTs were identified as being at low risk of bias within these categories,^16^, ^19^, ^23^, ^24^, ^25^, ^32^, ^35^, ^36^, ^37^, ^38^, ^39^ when looking at the potential for “other sources” of bias, all were seen as being at high risk due to a number of factors: they offered other analgesics besides the study drugs, recruited women who were looking for IUC placement and, in some cases, there was overlap in the VAS pain scores between treatment and placebo groups or the VAS pain scores in both groups were low (<4).

## 4 DISCUSSION

Our updated review set out to identify whether new RCTs contributed to further knowledge regarding effective pain strategies for IUC placement and identified 13 RCTs describing pharmacological interventions that result in lower pain scores when compared with placebo or control groups.^16^, ^22^, ^24^, ^25^, ^27^, ^29^, ^30^, ^31^, ^32^, ^34^, ^35^, ^36^, ^37^ The majority of these interventions involve pre‐insertion local anesthesia with lidocaine preparations. There is a need, however, for standardization of doses of these local analgesic agents, not only to optimize pain relief but also to maximize safety by preventing spread to vaginal tissue.^31^

The studies described reinforce the message that pain with IUC placement is not confined to the insertion of the device; use of the tenaculum and the uterine sound can also contribute to an uncomfortable experience. Assessment of post‐placement pain was limited in the studies we reviewed yet this may be important to consider for two reasons. First, although lack of time may limit the achievement of optimal effect of oral or local analgesia for IUC placement, these agents may help in the relief of post‐placement pain. Second, post‐procedural pain may contribute to the memory of a painful experience and lead to the sharing of negative views when talking to other women about IUC.

Multiple factors limit the ability to compare outcomes in the studies described: study populations, randomization protocols, concealment procedures, types of IUC placed, timing of pain assessment, and dose, formulation, and timing of analgesia all varied and limited the external validity of the findings. Although the instrument to measure pain (10‐cm or 100‐mm VAS) was used consistently across the majority of studies, the different assessment points for pain (both during the placement procedure and immediately following), use of inconsistent cut‐offs, and the potential for different anchor points limit inter‐study comparison. Additionally, most of the studies evaluated pain in all women who are looking for IUC placement and it is well known that many women experience no pain or minimal pain at IUC placement.^4^, ^5^, ^6^

Authors of nine of the RCTs reviewed here described the differences in pain scores between the intervention (oral ketorolac, vaginal misoprostol, lidocaine [spray, gel or paracervical block] and LP cream) and the placebo/control as being clinically relevant.^16^, ^24^, ^27^, ^29^, ^30^, ^32^, ^35^, ^36^, ^37^, ^38^ The differences in pain scores within these studies ranged from 15 to 41 mm. A systematic review by Olsen et al^61^ suggests 17 mm as the minimum median effect size on the VAS to be considered clinically relevant but note that it can vary according to whether there are single or multiple measurements. Using a median effect size of ≥17 mm as a benchmark, use of lidocaine paracervical block, novel lidocaine gels, LP cream, and lidocaine spray, were shown to achieve clinically relevant reductions in pain at the time of IUC placement when compared with placebo/control.^27^, ^29^, ^30^, ^32^, ^35^, ^36^ There is a need for more studies that emphasize the importance of clinically relevant changes in the VAS pain evaluation or focus on and recruit only women with a history of pain or difficulty with IUC placement. Grouping the pain responses into mild (0‐3), moderate (4‐6) and severe (7‐10) could provide more useful and relevant comparisons of different interventions.

Our review summarizes the findings of multiple studies looking at factors that increase the risk of pain with IUC placement. The effect of existing anxiety and beliefs about expected pain on actual pain during placement should not be under‐estimated. Women with mood disorders, a history of sexual trauma, a previous negative reaction to vaginal examination, a previous IUC placement that was painful, or awareness of the potential for pain from a friend or relatives may be more anxious about placement. When looking at whether anticipated pain vs actual pain could be a barrier to use of IUC, Narayan et al^55^ suggested that women choosing IUC may expect placement to be more painful and experience greater levels of pain than those choosing other contraceptive methods. However, it did not appear to affect their willingness to recommend IUC to friends either immediately following placement or 6 months later. Nevertheless, identifying women who anticipate pain and using evidence‐based pain management strategies may help to improve their experience. New studies, which evaluate these strategies in different groups of women, could help to change the perception of IUC placement and encourage more women to select IUC as a method.

### 4.1 Areas of potential future research

In addition to the ongoing need to evaluate pain‐relieving strategies in a systematic, validated way, there are several potential areas for future research, such as the impact of pain‐relieving strategies on the severity and duration of post‐placement pain and timing of administration of pre‐placement analgesia. For example, optimum administration time needed to achieve peak plasma concentrations of ketorolac (2 hours ahead of placement) was described as impractical for IUC placement in a busy outpatient setting by the authors.^16^

A number of studies described clinically relevant effects in placement‐related pain when using lidocaine gel or spray, or LP cream,^29^, ^31^, ^32^, ^35^, ^36^, ^37^ including novel formulations designed to maximize retention in the cervical canal. However, the practical benefit of local application of lidocaine may be limited by the length of time required for lidocaine to take effect in a busy clinic (3 minutes for spray; 7 minutes with the speculum in place for LP cream)^35^, ^36^ and contribute to existing anxiety around the procedure. Although only a small study, the significant reduction in placement‐related pain with a self‐administered, dual‐response lidocaine gel, compared with placebo, described by Abd Ellah et al^29^ showed the potential for the adaptation of existing agents to overcome limitations with administration.

Given that size and flexibility of the IUC can affect pain during placement, using smaller inserters and devices is likely to improve the experience for many women. The studies describing cervical priming showed that a moderate reduction in pain with IUC placement can be achieved with the use of vaginal misoprostol when compared with placebo.^22^, ^24^, ^25^ These studies also showed that misoprostol increased the likelihood of successful IUC placement and eased the procedure from a healthcare professional perspective in women who had a history of cesarean delivery or were nulligravidas. A study by Bahamondes et al^62^ also showed that misoprostol can increase the likelihood of successful IUC placement after previous insertion failure when compared with placebo: the risk ratios of successful placements in the evaluable population and the intent‐to‐treat population (95% CI) were 1.41 (8.2‐43.0) and 1.32 (0.3‐36.9), respectively. However, the side effect of pain caused by misoprostol‐induced uterine contractions remains an important consideration and may require adjunctive treatment with NSAIDs.

The previous literature review highlighted a lack of studies investigating the potential for nonpharmacological strategies, including cognitive treatment approaches, to minimize the pain and anxiety associated with IUC placement.^11^ Our updated review found no studies evaluating the use of cognitive treatment in this area despite growing recognition of the links between psychological factors and pain experience.^63^ Although we found evidence of a significant and clinically relevant reduction in placement‐related pain with the use of ultrasound to guide placement when compared with control (non‐guided technique),^46^ pain experience appears to be unaffected by the type of forceps used.^3^, ^43^, ^47^ Research into this area, therefore, remains important.

In conclusion, the number of publications found in the updated literature review indicates that the desire to identify ways to minimize pain with IUC placement remains an important goal. Evidence of clinically relevant effects on the experience of pain with pre‐placement analgesia when compared with placebo or controls is reported in studies evaluating different lidocaine preparations but routine use remains a subject for debate. Novel formulations of lidocaine gel, designed to ease administration, minimize leakage or prolong retention, appear particularly promising and further evaluation of these preparations in women who experience difficulty with placement would be a useful next step in achieving a more positive interpretation of the findings. Although placement‐related pain, insertion difficulties, and failures are uncommon, some women may be anxious or be at greater risk of painful placement and would, therefore, benefit from pain relief. Using an individual approach, guided by factors predictive of an increased risk of experiencing pain with IUC placement, could help to improve the experience for women. It may also contribute to the identification of effective, tailored strategies for routine use. In the modern era of medicine, our inability to recommend any positive treatment for pain relief with IUC placement creates professional discomfort. Further studies that use consistent approaches to timing of pain assessment could help to identify strategies to minimize the experience of pain and change the perception of IUC placement. In doing so, this may encourage more women to choose IUC as their contraceptive method.

## CONFLICT OF INTEREST

This publication and its content are solely the responsibility of the authors. KGD has participated in advisory board meetings on contraception and been the PI of clinical trials on IUS and other contraceptives, received honorarium for presentations on contraception for Bayer AG, MSD/Merck, HRA‐Pharma, Exeltis, Natural Cycles, and Mithra. These potential conflicts of interest have been reviewed and managed by KI. AdSS has participated as a member of Advisory Boards for Bayer AG. JTJ has received payments for consulting and research support from Bayer AG, Abbvie, Merck, HRA Pharma, Sebela, and the Population Council; consulting only from Cooper Surgical; and research support only from Daré, Mithra, and Medicines360. These companies and organizations may have a commercial or financial interest in the results of this research and technology. These potential conflicts of interest have been reviewed and managed by OHSU. TP has participated in Advisory Boards for Bayer AG and Shionogi Ltd. She has given presentations on Intrauterine Contraception and counseling and is a member of the INTRA Group. LB participated in advisory board meetings on contraception for Bayer AG and for MSD/Merck. IM and MR have nothing to disclose.
